# Outdoor Activities Associated with Lower Odds of SARS-CoV-2 Acquisition: A Case–Control Study

**DOI:** 10.3390/ijerph19106126

**Published:** 2022-05-18

**Authors:** Tommaso C. Bulfone, Cinthia Blat, Yea-Hung Chen, George W. Rutherford, Luis Gutierrez-Mock, Andrea Nickerson, Laura Buback, Susie Welty, Karen Sokal-Gutierrez, Wayne T. A. Enanoria, Michael J. A. Reid

**Affiliations:** 1 School of Medicine, University of California, San Francisco, CA 94143, USA; tommaso.bulfone@ucsf.edu; 2 School of Public Health, University of California, Berkeley, CA 94704, USA; ksokalg@berkeley.edu; 3 Institute for Global Health Sciences, University of California, San Francisco, CA 94143, USA; cinthia.blat@ucsf.edu (C.B.); george.rutherford@ucsf.edu (G.W.R.); luis.gutierrezmock@ucsf.edu (L.G.-M.); andrea.d.nickerson@gmail.com (A.N.); laura.buback@ucsf.edu (L.B.); susie.welty@ucsf.edu (S.W.); 4 Department of Epidemiology and Biostatistics, University of California, San Francisco, CA 94143, USA; yea-hung.chen@ucsf.edu (Y.-H.C.); wayne.enanoria@sfdph.org (W.T.A.E.); 5 San Francisco Department of Public Health, San Francisco, CA 94102, USA; 6 Department of Medicine, Division of HIV, Infectious Diseases and Global Medicine, University of California, San Francisco, CA 94143, USA

**Keywords:** COVID-19, SARS-CoV-2, transmission, outdoor activity, outdoor parks

## Abstract

Access to recreational physical activities, particularly in outdoor spaces, has been a crucial outlet for physical and mental health during the COVID-19 pandemic. There is a need to understand how conducting these activities modulates the risk of SARS-CoV-2 infection. In this case–control study of unvaccinated individuals conducted in San Francisco, California, the odds of testing positive to SARS-CoV-2 were lower for those who conducted physical activity in outdoor locations (adjusted odds ratio [aOR]: 0.16, 95% confidence interval [CI]: 0.05, 0.40) in the two weeks prior to testing than for those who conducted no activity or indoor physical activity only. Individuals who visited outdoor parks, beaches, or playgrounds also had lower odds of testing positive to SARS-CoV-2 (aOR: 0.28, 95% CI: 0.11, 0.68) as compared with those who did not visit outdoor parks, beaches, or playgrounds. These findings, albeit in an unvaccinated population, offer observational data to support pre-existing ecological studies that suggest that activity in outdoor spaces lowers COVID-19 risk.

## 1. Introduction

The COVID-19 pandemic has caused widespread decreases in individual physical and recreational activity, particularly in outdoor spaces [1]. This resulted in widening health disparities, such as increased rates of mental health conditions [2] and chronic disease [3] in at-risk populations. While vaccination remains the cornerstone of prevention efforts, non-pharmaceutical interventions have an important role, especially as new mutations arise and vaccination coverage remains suboptimal. Effective non-pharmaceutical interventions include conducting physical, social, and recreational activities outdoors where the risk of aerosol transmission is presumably lower. However, more precise evidence is needed to determine which activities heighten or lower the risk of SARS-CoV-2 transmission outdoors.

Several ecological studies have observed an association between the availability of green space and reduced COVID-19 case rates [4,5,6,7,8,9]. However, this association has not yet been explored in an observational study that investigates outdoor risk at an individual level.

We conducted a case–control study to investigate whether outdoor physical and recreational activities were associated with SARS-CoV-2 transmission in the city and county of San Francisco (CCSF) from April to June 2021, when average daily cases ranged from 43 to 10 and the proportion of residents who were fully vaccinated increased from ~32% to ~65% [10]. At the time of the study, public parks and playgrounds were fully open with distancing requirements, and informal outdoor recreation was allowed for groups of up to 25 people with face coverings and social distancing [11]. In this unique time window, during which the majority of individuals were not yet vaccinated in San Francisco and before the spread of the more transmissible SARS-CoV-2 variants, we hypothesized that outdoor recreation might modulate odds of transmission for non-vaccinated individuals.

## 2. Materials and Methods

We identified participants from the San Francisco Department of Public Health surveillance database of SARS-CoV-2 test records during the study period. Using a case–control design, we recruited unvaccinated individuals aged 13 years and older who had tested positive for SARS-CoV-2 (“cases”), as well as a random sample of those who tested negative (“controls”). Given the narrow time window, we did not match for the date of the test result. The study was approved by the University of California, San Francisco, Committee on Human Research. Informed consent was required of all study participants, including participants younger than 18 years and their parents/guardians.

Exclusion criteria included having received ≥ 1 dose of a COVID-19 vaccine, residing outside of CCSF, living in a congregate setting (such as a long-term care facility, homeless shelter, or jail), or being unable to confirm birth date or test date. Cases were also excluded if they were unaware of their SARS-CoV-2 status or had not yet been interviewed by the health department’s case investigation team. To minimize exposure recall bias, we contacted subjects within three weeks of their test dates.

We attempted to reach all remaining eligible study participants by telephone and completed an interviewer-led, closed-ended questionnaire on activities during the two-week period prior to their test dates (Supplementary Table S1). Interviews assessed demographic factors and exposures related to physical and recreational activity (e.g., indoor/outdoor sports, venues for exercise, and types of exercise activity). Interviews were conducted in English, Spanish, Mandarin, and Tagalog.

We examined the sociodemographic characteristics of the cases and controls, and tested for differences using Pearson’s Chi-squared and Fisher’s exact tests. We then fitted logistic regression models to generate odds ratios for SARS-CoV-2 status by outdoor sport and outdoor park use, adjusting for potential confounding variables including age, race/ethnicity, ZIP code per capita income, and household occupancy. Household occupancy was defined by occupants per room: under-occupied was defined as fewer than one occupant per room, balanced was defined as one per room, and over-occupied was defined as more than one per room. In addition, we performed exploratory interaction analyses to test for the effect modification of SARS-CoV-2 status and outdoor sport and park use by household occupancy and ZIP code per capita income. ZIP code estimated per capita income was obtained as a computed average from the publicly available American Community Survey 2019 Census Dataset [12] and dichotomized according to the cutoff for “very low income” in San Francisco defined by the United States Department of Housing and Urban Development [13]. Finally, to test for potential bias due to the inclusion of adolescents (ages 13–17) in the sample, sensitivity analyses were conducted by restricting the sample to adults only (ages 18+).

All statistical analyses were conducted using R version 4.0.5 (R Computing Group, Vienna, Austria) [14].

## 3. Results

Among the sampled participants from a pool of 267,464 records, 982 cases and 1433 controls were reached by phone. In total, 139 cases and 35 controls consented, met the eligibility criteria, and completed questionnaires. The median age for the sample was 31.5 years (interquartile range [IQR] 25–43), 51% were male, and 35% identified as Latinx.

In the two weeks prior to testing, 43% had participated in an outdoor sport and 42% had visited an outdoor park, beach, or playground. In the bivariate analysis, cases and controls differed with respect to age (*p* = 0.002) and race/ethnicity (*p* = 0.04) (Table 1). Cases were less frequent among youth aged 13–17 years (4.3% cases vs. 22.9% controls) and more frequent among subjects aged 18–29 years (36.7% cases vs. 14.3% controls) and those who identified as Latinx (38.6% cases vs. 20.6% controls). Gender distribution was similar in cases and controls (female gender: 48.9% cases vs. 45.7% controls).

In the multivariable analysis, cases were less likely to engage in outdoor sports and less likely to use outdoor parks, beaches, or playgrounds, regardless of activity (Table 2). There was no association between case status and household occupancy or estimated per capita income by ZIP code (data not shown).

Interaction analyses found no statistically significant effect modification of SARS-CoV-2 status and outdoor sport and park use by household occupancy or ZIP code per capita income (*p* ≥ 0.25 for all interactions) (Supplementary Tables S2–S5). Additional sensitivity analyses which restricted participants to adults ≥ 18 years yielded similar findings to Table 2 (*p*-values, model a: <0.001, model b: 0.03).

## 4. Discussion

In this case–control study conducted among unvaccinated individuals in San Francisco, California, from 7 April to 8 June 2021, we found that the odds of testing positive for SARS-CoV-2 were significantly lower for those who conducted physical activity in outdoor locations in the two weeks prior to testing than those who conducted no activity or indoor physical activity only. The protective effect of outdoor locations was further highlighted by the association between low odds of SARS-CoV-2 infection and visiting outdoor parks, beaches, or playgrounds.

These findings, based on individual-level observational data, support ecological studies that found an association between the availability of green space and reduced COVID-19 case rates [4,5,6,7,8,9]. We hypothesize three reasons why outdoor use might have decreased the odds of infection in our study.

First, recreational activities in outdoor spaces may prevent SARS-CoV-2 transmission insofar that they offer safe, healthy outlets that may replace other, riskier activities indoors. This relationship might partly explain the disproportionate burden of COVID-19 on marginalized populations in lower-income, higher-density urban settings, who historically have little or no access to safe and clean green spaces [15]. Even though low income by ZIP code did not modify the association of outdoor use and decreased odds of COVID-19 in our study, other studies have found that outdoor access in minority, low-income populations was associated with lower COVID-19 rates [4,5].

Second, individuals who reported any physical activity in outdoor spaces in the two weeks prior to testing might be healthier overall. Green space use, which has been shown to have decreased individual stress levels during the pandemic [16], and consistent physical activity, which has been associated with a reduced risk of severe COVID-19 [17], could both contribute to biological protective factors against SARS-CoV-2 infection and severity.

Lastly, high vaccine uptake in San Francisco (roughly 65% [18] of all residents by the end of the study period, including children, were fully vaccinated at the end of the study) might have affected how transmission spread in certain contexts. If the majority of individuals who took part in activities with higher perceived risk, such as physical and recreational activity, were vaccinated, the risk of infection would have decreased among individuals practicing those activities.

In theory, the local policies with respect to social gatherings in place at the time of the study could also have influenced our findings. Guidance from CCSF at the time limited informal indoor gatherings to 12 people but for outdoor recreation, the limit was up to 25 people [11]. As such, we might have expected to see greater risk in outdoor venues where more people could congregate, although this was not the case. Concurrently, some high-risk indoor recreation locations, such as schools, remained closed.

This study has several limitations. The study has a small sample size due to the diminishing COVID-19 case count during data collection, which made it difficult to recruit eligible participants. The study was conducted before the newer wave of the substantially more transmissible COVID-19 variants, such as B.1.617.2 (Delta) and B.1.1.529 (Omicron). Additionally, challenges in the recruitment of controls due to increased vaccination rates during the time of the study and the limited success of COVID-19-safe recruitment methods (e.g., telephone) limited the size of the control group, regardless of oversampling controls in the study participant selection phase. Selection bias might have affected the results, as people who did not participate in the study might have been different from individuals included in this study, for example, due to different lifestyle factors. Moreover, we acknowledge that the regression models may not have adjusted for all possible confounders. Interviewees might have been prone to recall bias, as awareness of their COVID-19 test result might have influenced their responses. Finally, our questionnaire did not include the duration and frequency of outdoor activities.

## 5. Conclusions

In this case–control study of unvaccinated individuals, the adjusted odds of testing positive for SARS-CoV-2 were 84% lower among individuals who conducted outdoor recreational physical activity in the two weeks prior to testing than those who conducted no activity or indoor activity only. The adjusted odds of testing positive for SARS-CoV-2 were 72% lower among individuals who visited outdoor parks in the two weeks prior to testing than who did not. These findings support pre-existing ecological studies which suggest that proximity to green spaces and activity in outdoor spaces might lower the overall COVID-19 risk. Efforts to increase green space and its use, particularly in areas with decreased access, are therefore urgent: equity in access to outdoor space may not only be crucial for individual physical and mental health, but also may be an important protective factor in decreasing the spread of respiratory infections, further contributing to health of communities overall.

## Figures and Tables

**Table 1:** Demographic characteristics.

Characteristic	Cases (*n* = 139)	%	Controls (*n* = 35)	%	*p*-Value
Age, yrs					0.002
13–17	6	4.3	8	22.9	
18–29	51	36.7	5	14.3	
30–54	68	48.9	19	54.3	
55 and over	14	10.1	3	8.6	
Race/ethnicity					0.04
Asian	17	12.9	5	14.7	
Black or African-American	27	20.5	5	14.7	
Latinx	51	38.6	7	20.6	
White	22	16.7	14	41.2	
Other	15	11.4	3	8.8	
Gender					0.85
Female	67	48.9	16	45.7	
Male	69	50.4	19	54.3	
Genderqueer or non-binary	1	0.7	0	0.0	
Sexual orientation					0.05
Heterosexual or straight	123	93.2	20	80.0	
LGBTQ	9	6.8	5	20.0	
Missing *	7		10		
Household occupancy °					0.94
Balanced/under-occupied	68	48.9	18	51.4	
Over-occupied	71	51.1	17	48.6	
Estimated per capita income by ZIP code					0.88
High	72	51.8	17	48.6	
Low	67	48.2	18	51.4	

* Participants below the age of 18 were not asked about their sexual orientation. ° Under-occupied—fewer than one occupant per room, balanced—one occupant per room, over-occupied—more than one occupant per room.

**Table 2:** Adjusted odds ratios for COVID-19 by outdoor sport and park use.

Group *	Cases *n* = 139 (%)	Controls *n* = 35 (%)	aOR	95% CI	*p*-Value
Outdoor sport participation					
Any outdoor sport	49 (35.8)	24 (70.6)	0.16 ^a^	0.05, 0.40 ^a^	<0.001
No sport/indoor only sport	88 (64.2)	10 (29.4)	ref		
Outdoor park use					
Visit to park, beach, or playground	52 (38.0)	21 (60.0)	0.28 ^b^	0.11, 0.68 ^b^	0.006
No visit to park, beach, or play ground	85 (62.0)	14 (40.0)	ref		

Abbreviations: aOR, adjusted odds ratio; CI, confidence interval; ref, reference. ^a^ Model included household occupancy, ZIP code estimated per capita income, sport participation, age and race/ethnicity. ^b^ Model included household occupancy, ZIP code estimated per capita income, park use, age and race/ethnicity. * In additional sensitivity analyses, study participants were similar to non-respondents by gender and per capita income by ZIP code. Non-respondents tended to be younger than the study participants.

## Data Availability

Not applicable.

## Supplementary Materials

The following supporting information can be downloaded at: https://www.mdpi.com/article/10.3390/ijerph19106126/s1. Table S1: Selected questionnaire questions. Table S2: Adjusted odds ratios for COVID-19 by sport participation and household occupancy. Table S3: Adjusted odds ratios for COVID-19 by outdoor park use and household occupancy. Table S4: Adjusted odds ratios for COVID-19 by sport participation and ZIP code median per capita income. Table S5: Adjusted odds ratios for COVID-19 by outdoor park use and ZIP code median per capita income.
