# Supplementary material for: Outdoor Activities Associated with Lower Odds of SARS-CoV-2 Acquisition: A Case–Control Study
Source: Int J Environ Res Public Health. 2022 May 18;19(10):6126. doi: 10.3390/ijerph19106126 (PMC9141379; doi:10.3390/ijerph19106126)
Supplement: Supplementary file 1 [file ijerph-19-06126-s001.zip › ijerph-1661061-supplementary.pdf]

Supplementary Tables

Table S1: Study Questionnaire – Selected Relevant Questions

In the two weeks prior to testing, did you ever:<br>(Select all that apply)	Visit an indoor gym or attend an indoor fitness class	
	Engage in any organized sports or recreation in an indoor setting. Examples of sports include: badminton, bowling, dancing, ice skating, physical training, tennis, track and field, basketball, martial arts, and wrestling	
	Engage in any organized sports in an outdoor setting (including an outdoor gym or outdoor fitness class)	
	Engage in any recreation in an outdoor setting (including running, hiking, biking or other)	
	Swim at a public swimming pool or marina	
	Go to a public playground	
	Go to an outdoor park or beach	
Specific sports questions:	Did you play frisbee indoors or outdoors?	Indoors
		Outdoors
		Decline to answer
	Did you do yoga or pilates indoors or outdoors?	Indoors
		Outdoors
		Decline to answer
	Did you do Zumba indoors or outdoors?	Indoors
		Outdoors
		Decline to answer
	Did you do HIIT indoors or outdoors?	Indoors
		Outdoors
		Decline to answer
	Did you do bocce ball/lawn bowling/bowling indoors or outdoors?	Indoors
		Outdoors
		Decline to answer
	Did you walk, run, or hike indoors or outdoors?	Indoors
		Outdoors
		Decline to answer
	Did you do stationary cycling indoors or outdoors?	Indoors
		Outdoors
		Decline to answer
	Did you swim indoors or outdoors?	Indoors
		Outdoors
		Decline to answer
	Did you play badminton indoors or outdoors?	Indoors
		Outdoors
		Decline to answer

	Did you play no contact dancing indoors or outdoors?	Indoors
		Outdoors
		Decline to answer
	Did you partner dance indoors or outdoors?	Indoors
		Outdoors
		Decline to answer
	Did you do physical training indoors or outdoors?	Indoors
		Outdoors
		Decline to answer
	Did you play singles tennis indoors or outdoors?	Indoors
		Outdoors
		Decline to answer
	Did you play doubles tennis indoors or outdoors?	Indoors
		Outdoors
		Decline to answer
	Did you play golf indoors or outdoors?	Indoors
		Outdoors
		Decline to answer
	Did you play baseball/softball indoors or outdoors?	Indoors
		Outdoors
		Decline to answer
	Did you do cheerleading indoors or outdoors?	Indoors
		Outdoors
		Decline to answer
	Did you play dodgeball indoors or outdoors?	Indoors
		Outdoors
		Decline to answer
	Did you play field hockey indoors or outdoors?	Indoors
		Outdoors
		Decline to answer
	Did you do gymnastics indoors or outdoors?	Indoors
		Outdoors
		Decline to answer
	Did you play kickball indoors or outdoors?	Indoors
		Outdoors
		Decline to answer
	Did you play soccer indoors or outdoors?	Indoors
		Outdoors
		Decline to answer
	Did you play football indoors or outdoors?	Indoors
		Outdoors
		Decline to answer
	Did you play basketball indoors or outdoors?	Indoors
		Outdoors
		Decline to answer

	Did you play ice hockey indoors or outdoors?	Indoors
		Outdoors
		Decline to answer
	Did you do martial arts indoors or outdoors?	Indoors
		Outdoors
		Decline to answer
	Did you play water polo indoors or outdoors?	Indoors
		Outdoors
		Decline to answer
	Did you row/crew indoors or outdoors?	Indoors
		Outdoors
		Decline to answer
	Did you wrestle indoors or outdoors?	Indoors
		Outdoors
		Decline to answer
	Did you do (the other activity) indoors or outdoors?	Indoors
		Outdoors
		Decline to answer

Table S2. Adjusted odds ratios for COVID-19 by sport participation and household occupancy [a].

**Group**	Case	Control	aOR	95%CI	p-value
Over-occupied					
Outdoor only/Combined sport	22 (31.0)	10 (62.5)	**0.19**	**0.05, 0.75**	**0.006**
No sport/Indoor only sport	49 (69.0)	6 (37.5)	ref		
Balanced/Under-occupied					
Outdoor only/Combined sport	27 (40.9)	14 (77.8)	**0.12**	**0.03, 0.55**	**0.02**
No sport/Indoor only sport	39 (59.1)	4 (22.2)	ref		
household occupancy and sport participation interaction p-value = 0.66					

Abbreviations: aOR, adjusted odds ratio; CI, confidence interval.

[a] Model included household occupancy, sport participation, sport by household occupancy interaction term,
ZIP code median per capita income, age and race/ethnicity

Table S3. Adjusted odds ratios for COVID-19 by outdoor park use and household occupancy [a].

**Group**	Case	Control	aOR	95%CI	p-value
Over-occupied					
Outdoor park use	23 (32.9)	9 (52.9)	0.35	0.10, 1.21	0.10

No outdoor park use	47 (67.1)	8 (47.1)	ref		
Balanced/Under-occupied					
Outdoor park use	29 (43.3)	12 (66.7)	**0.22**	**0.06, 0.82**	**0.02**
No outdoor park use	38 (56.7)	6 (33.3)	ref		
household occupancy and outdoor park use interaction p-value = 0.61					

Abbreviations: aOR, adjusted odds ratio; CI, confidence interval.
[a] Model included household occupancy, sport participation, sport by household occupancy interaction term,
ZIP code per capita income, age and race/ethnicity

Table S4. Adjusted odds ratios for COVID-19 by sport participation and ZIP code median per capita income [a].

**Group**	Case	Control	aOR	95%CI	p-value
Low					
Outdoor only/Combined sport	18 (27.3)	12 (70.6)	**0.09**	**0.02, 0.37**	**<0.001**
No sport/Indoor only sport	48 (72.7)	5 (29.4)	ref		
High					
Outdoor only/Combined sport	31 (43.7)	12 (70.6)	0.27	0.07, 1.04	0.06
No sport/Indoor only sport	40 (56.3)	5 (29.4)	ref		
ZIP code per capita income and sport participation interaction p-value = 0.25					

Abbreviations: aOR, adjusted odds ratio; CI, confidence interval.
[a] Model included ZIP code per capita income, park use, park use by ZIP code median per capita income cross-product term, household occupancy, age and race/ethnicity

Table S5. Adjusted odds ratios for COVID-19 by outdoor park use and ZIP code median per capita income [a].

**Group**	Case	Control	aOR	95%CI	p-value
Low					
Outdoor park use	17 (25.8)	11 (61.1)	**0.19**	**0.05, 0.69**	**0.01**
No outdoor park use	49 (74.2)	7 (38.9)	ref		
High					
Outdoor park use	35 (43.7)	10 (70.6)	0.42	0.12, 1.50	0.18
No outdoor park use	36 (56.3)	7 (29.4)	ref		
ZIP code per capita income and outdoor park use interaction p-value = 0.39					

Abbreviations: aOR, adjusted odds ratio; CI, confidence interval.

[a] Model included ZIP code per capita income, park use, park use by ZIP code median per capita income cross-product term, household occupancy, age and race/ethnicity
